# Milk Purity: The Distributor's View

**Published:** 1922-05

**Authors:** Reginald Butler


### MILK PURITY: THE DISTRIBUTOR'S VIEW.

[Sir Reginald Butler, who was recently made a baronet for his services to agriculture, is an expert on milk questions in this country, and we are glad to be able to give his views on this question.—Ed.]

In reply to a question as to the relation between the recent reduction in price of milk and its value as a food, Sir Reginald Butler stated that the present tidal wave of milk should prove to be a blessing to the community. "Milk contains all the nutritious constituents required by the body, including protein, fats and carbo-hydrates, as well as vitamines, those mysterious substances which promote growth and are proved to be necessary for maintaining the body in a healthy condition. Milk is to-day the cheapest and the most valuable food available. The Astor report stated that the consumption of milk in the United Kingdom was much lower than was desirable in the national interest. The glut of milk has enabled the price to the consumer in London to be reduced to 2½d. per pint, and this will in all probability be maintained for some time to come. It is expected that there will be a daily surplus of considerably over half a million gallons during the next three months, and one way by which the public can serve both their own interests and that of the farmers who are inevitably suffering from this glut is to increase the consumption. If every child under three years of age drank the amount of milk declared to be necessary by the medical profession, the surplus would be exhausted and there would be little milk available for the adult public."

"What is being done to increase the cleanliness of milk?"

"I can deal with this question only from the point of view of the distributor, but certainly the large wholesale firms realise that the first essential of efficient milk distribution is to ensure that the products of the dairy farmer reach the consumer in a pure and wholesome condition. In this question of cleanliness, the interest of the farmer is identical with that of the distributor, who in many cases collects the milk from the farmer and is responsible for it until it is left at the door of the householder. At every stage we are endeavouring to guarantee, in spite of many difficulties, that all milk sold is pure, clean, sweet and of the requisite quality. The cleaner the milk, the more rapidly the rate of consumption will increase, ensuring a better market for the farmers' supplies.

"This sounds simple enough to those who do not understand the various stages through which milk has to pass on its way from the producer to the consumer. The milk has to be collected from many farmers and over wide areas, on motor-lorries which convey it to different creameries. There the milk is cleaned, pasteurised and cooled. On its arrival at the great London termini it is collected for delivery to the dairies' central depots. Milk received at these depots is subject to careful analytical inspection by experts. It is to the high standard rigorously insisted upon at these depots that the quality and the cleanliness of London's milk supply and its freedom from bacteriological taint is very largely due. Where milk is bottled and sealed, there is, of course, a guarantee that no contamination will take place before the bottle is opened by a householder. But those responsible for the wholesale distribution of milk not placed in bottles are endeavouring to provide safeguards for the milk throughout its journey."

"Do you think that improvements are taking place?"

"Yes, great improvements have taken place during the past few years, and better sanitary arrangements now prevail at a great number of dairy farms. But there is no article of food so susceptible of taint as milk. Milk that leaves the farm dirty is quickly contaminated. I am of opinion that the high standard set by Grade "A" milk, for which a Government certificate is given, is assisting the whole trade. In London the public demand now exceeds the supply, and those who have taken the trouble to go in for up-to-date methods are being rewarded. But Grade "A" milk is inevitably expensive. There must be co-operation between the producer and all those associated with him to ensure the most scrupulous cleanliness in the production and handling of milk and its delivery in a wholesome condition, at the lowest possible price, to the consumer. Even then our task is not finished, for milk left uncovered on the kitchen table, or even on the doorstep, may become soured although it has been delivered fresh. There must be an alliance of producer, distributor and consumer if we are to achieve victory in the campaign for cleaner milk."

